# Computer-Based Diagnosis of Celiac Disease by Quantitative Processing of Duodenal Endoscopy Images

**DOI:** 10.3390/diagnostics13172780

**Published:** 2023-08-28

**Authors:** Adriana Molder, Daniel Vasile Balaban, Cristian-Constantin Molder, Mariana Jinga, Antonin Robin

**Affiliations:** 1 Center of Excellence in Robotics and Autonomous Systems, Military Technical Academy Ferdinand I, 050141 Bucharest, Romania; 2 Internal Medicine and Gastroenterology, Central Military Emergency University Hospital, Carol Davila University of Medicine and Pharmacy, 030167 Bucharest, Romania; 3 Department of Electronics and Digital Technologies, Polytech Nantes, 44300 Nantes, France

**Keywords:** celiac disease, endoscopic images, machine learning, deep learning

## Abstract

Celiac disease (CD) is a lifelong chronic autoimmune systemic disease that primarily affects the small bowel of genetically susceptible individuals. The diagnostics of adult CD currently rely on specific serology and the histological assessment of duodenal mucosa on samples taken by upper digestive endoscopy. Because of several pitfalls associated with duodenal biopsy sampling and histopathology, and considering the pediatric no-biopsy diagnostic criteria, a biopsy-avoiding strategy has been proposed for adult CD diagnosis also. Several endoscopic changes have been reported in the duodenum of CD patients, as markers of villous atrophy (VA), with good correlation with serology. In this setting, an opportunity lies in the automated detection of these endoscopic markers, during routine endoscopy examinations, as potential case-finding of unsuspected CD. We collected duodenal endoscopy images from 18 CD newly diagnosed CD patients and 16 non-CD controls and applied machine learning (ML) and deep learning (DL) algorithms on image patches for the detection of VA. Using histology as standard, high diagnostic accuracy was seen for all algorithms tested, with the layered convolutional neural network (CNN) having the best performance, with 99.67% sensitivity and 98.07% positive predictive value. In this pilot study, we provide an accurate algorithm for automated detection of mucosal changes associated with VA in CD patients, compared to normally appearing non-atrophic mucosa in non-CD controls, using histology as a reference.

## 1. Introduction

Celiac disease (CD) is among the most frequent chronic digestive diseases worldwide but is severely underdiagnosed [1]. It is an autoimmune, systemic, malabsorptive disease that primarily affects the small bowel, leading to a crypt hyperplastic, atrophic injury of the mucosa, in response to dietary intake of gluten. This occurs in genetically susceptible individuals and affects approximately 1% of the population worldwide [2]. Currently, adult CD diagnosis relies on the combination of serological testing and upper gastrointestinal endoscopy with small bowel biopsies, in order to detect the atrophic gluten-induced mucosal damage [3]. In pediatrics, CD diagnosis can be made based on serology only [4], and this has fueled a growing interest in a biopsy-avoiding diagnostic strategy in adults also [5,6]. There are several anticipated benefits of a no-biopsy diagnosis in CD: lowering costs (by eliminating the need for biopsy sampling, biopsy processing, and histopathology analysis), avoiding the wearing and tearing of the working channel of the scope, reducing the procedural time and exposure to sedation and its associated side effects, and not least avoiding the pitfalls of histology reported in CD diagnosis [7,8]. In this setting, several studies have looked at the association between villous atrophy (VA) proven by histological analysis and specific changes in the small bowel mucosa, the so-called endoscopic markers of VA [9] (see Table 1).

In fact, the detection of VA on endoscopic examinations completed for CD-unrelated indications is considered an opportunity for the detection of clinically unsuspected CD [10]. Considering the large number of endoscopic examinations worldwide, along with the availability of open-access endoscopy in some services, recognition of VA markers becomes of paramount importance in potentially improving the diagnostic rate of CD. Some authors have shown that a significant proportion of patients with CD had a previous recent endoscopic examination that might have missed the subtle changes in the small bowel mucosa and failed to provide an early diagnosis [11]. A potential role for computer-aided detection of mucosal changes is foreseen in this setting, as already validated for other pathologies [12].

**Figure 1:**
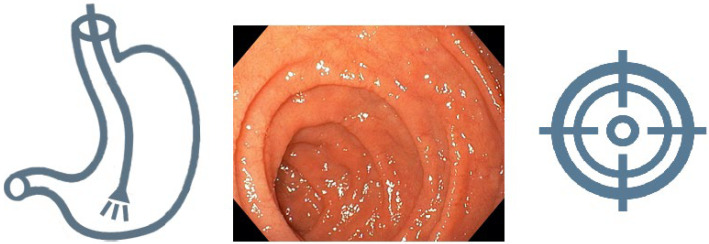
Unmasking clinically unsuspected CD by opportunistic detection of VA on endoscopy.

There is already solid evidence regarding the quantitative processing of capsule endoscopy images for CD diagnosis [13], but videocapsule examination is far less commonly used than upper gastrointestinal endoscopy. Endoscopy is used both in suspicion of CD and for confirmation of diagnosis [3], but the major window of opportunity is represented by examinations conducted for non-CD related reasons, where the detection of VA by computer-aided diagnosis could significantly improve CD case findings (see Figure 1).

Artificial intelligence (AI) has undoubtedly revolutionized practice in several medical fields, including gastroenterology. Computer-aided image analysis has already been explored for endoscopic procedures, ultrasound images, and histology slides in both luminal and hepato-bilio-pancreatic pathologies. In the field of endoscopy, there are abundant data on AI applications for colonoscopy, a procedure well recognized to be operator-dependent, for polyp detection and diagnosis, and also bowel cleansing, and in this setting, AI techniques have proven to improve quality indicators of examinations [14]. In addition to the use in colorectal cancer screening programs, in the lower gastrointestinal tract, there are also AI applications for inflammatory bowel disease, both Crohn’s disease and ulcerative colitis. Concerning the upper gastrointestinal tract, there are validated algorithms for Barrett’s esophagus [15], chronic atrophic gastritis [16], esophageal and gastric cancer [17], and for the small bowel, there is AI assistance for the detection of bleeding on capsule endoscopy software. Not least, we have significant data on AI for liver disease—hepatocellular carcinoma, liver fibrosis, and pancreatic pathology—both benign (acute pancreatitis) and malignant (pancreatic cancer) [17]. The benefit of using AI in medicine is not only about improving diagnosis and detection but also saving time and providing faster and wider access to healthcare services by optimizing resource use.

Most common AI classification algorithms have been evaluated in order to retain the ones that provide the most accuracy for further studies. As there is no “golden bullet” solution for medical image classification, and while deep learning algorithm performances are highly sensitive to the training image database, no a priori presumption has been made related to the best classifier.

AI algorithms have already been validated for detecting CD in capsule endoscopy images and also for automated CD diagnosis on biopsy slides [13,18]. We aimed to assess if computer processing of duodenal images captured during endoscopy would be accurate in detecting mucosal changes associated with VA in CD patients.

## 2. Materials and Methods

**Figure 2:**
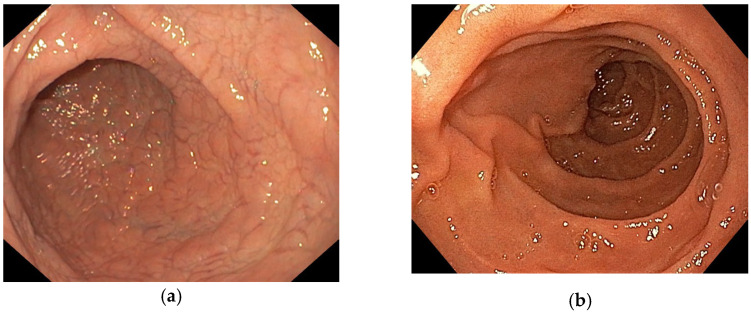
Comparative endoscopic images from (**a**) a CD confirmed and (**b**) a control patient.

We performed an observational study including patients, with clinical suspicion of CD, who were tested by serology (IgA tissue transglutaminase and total serum IgA, IgG tissue transglutaminase in case of IgA deficit) and who also underwent upper gastrointestinal endoscopy with multiple biopsies from the bulb and distal duodenum. Tissue transglutaminase antibodies were assessed using Celikey assay (Thermo Fisher Scientific) and endoscopy was performed using high-definition scopes from Olympus (Tokyo, Japan). Histopathology results were reported according to Marsh–Oberhuber classification [19]. CD diagnosis was made according to currently available guidelines [3,20]. Based on the results of serology and duodenal histopathology, patients were diagnosed as CD or non-CD. All CD patients had atrophic mucosal injury in the distal duodenum (Marsh 3), while non-CD controls had Marsh 0-1 histology. During endoscopy, at least one photo was captured from the distal duodenum, before taking the biopsy samples. Caption of the images was performed with the probe positioned in the second duodenum, upon straightening of the scope during the shortening maneuver (see Figure 2). We excluded patients with diagnostic uncertainties. Photos were transferred anonymously for image processing. Classification of images after applying an automated processing algorithm was then compared to histology for each corresponding patient. All patients included in the analysis signed informed consent for using anonymized medical data, including endoscopy images, for research purposes.

In order to detect VA on endoscopy-captured images, we used machine learning (ML) and deep learning (DL) algorithms. It has been shown that ML performs on par with medical experts [21] and software applications are starting to be certified for clinical use [22,23]. Some papers have proposed methods to eliminate degradations such as noise, reflections, blurring, and scaling due to weak illumination and downsize sensors [24,25,26].

**Figure 3:**
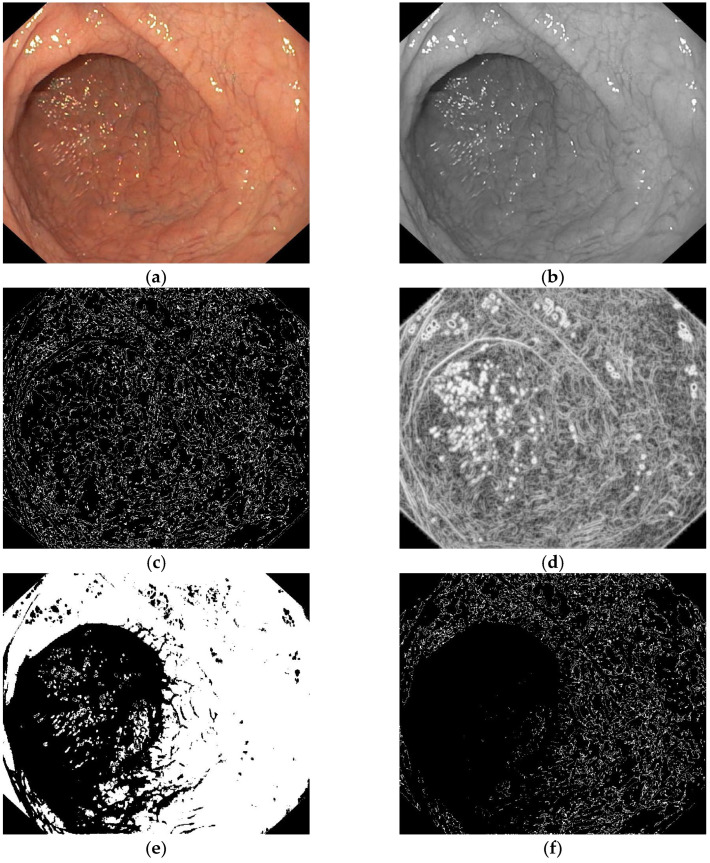

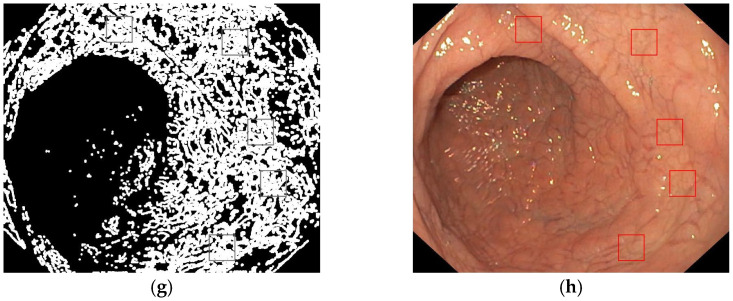
Automatic patches extraction process: (**a**) original CD image, (**b**) gray level image, (**c**) entropy filtered image, (**d**) binarization of the entropy filtered image, (**e**) binarization of the gray level image, (**f**) logic AND between binary images, (**g**) dilatation and patches selection, (**h**) patches on original CD image.

**Figure 4:**
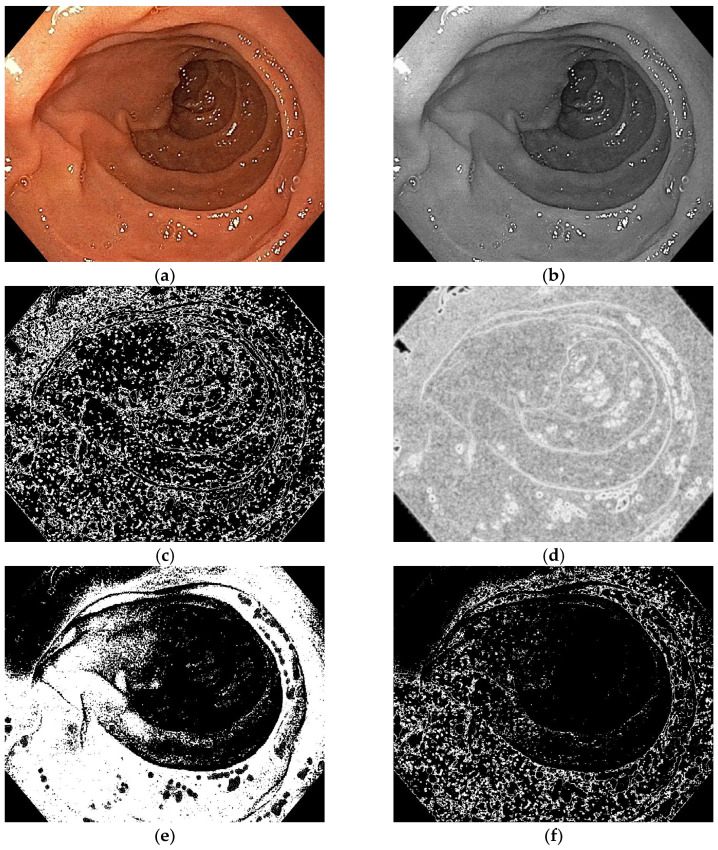

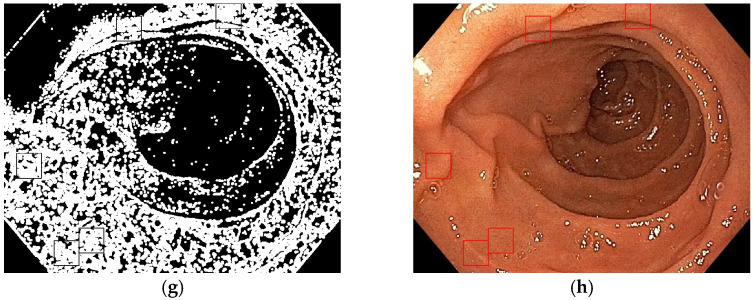
Automatic patches extraction process: (**a**) original control image, (**b**) gray level image, (**c**) entropy filtered image, (**d**) binarization of the entropy filtered image, (**e**) binarization of the gray level image, (**f**) logic AND between binary images, (**g**) dilatation and patches selection, (**h**) patches on original control image.

This paper proposes a new model of automatic computerized method of image processing in which artifacts caused by the presence of air bubbles, residues, or secretions in the duodenum are excluded so that they do not influence the final decision of the classifier. We implemented an algorithm that extracts clean image frames from the image captured during endoscopy. These patches exclude areas containing artifacts, too bright or too dark areas present in the endoscopic image. Figure 3 and Figure 4 show the automatic patch extraction process on CD and control images, respectively:

- Entropy filter applied on the gray level image;
- Binarization of the entropy-filtered image with high and low thresholds;
- Binarization of the gray level image using two high and low thresholds;
- Logic AND between binary images and dilatation;
- Patches selection according to the final binary image and the gray level range.

After generating patches, we assessed several ML algorithms. A total of 23 types of ML methods were implemented, of which we selected the three most relevant: weighted K-nearest neighbors (WKNN), boosted trees, and bagged trees. Weighted kNN is a modified version of the classic k nearest neighbor algorithm that overcomes the drawbacks of the choice of the parameter k. By using a kernel function such as the squared inverse distance function, known samples that are closer to the query are given more importance than samples that are situated farther. Boosted trees are classifiers that combine two techniques (a decision tree and a boosting method) in order to improve accuracy. Bagged trees are methods to reduce the variance of the learning method using the same type of prediction.

For the selected methods we used the following parameters:

- WKNN: K = 10, Euclidean distance, squared inverse distance weight;
- Boosted trees: AdaBoost ensemble method, 20 maximum splits, 30 learners, learning rate = 0.1;
- Bagged trees: bag ensemble method, 536 maximum splits, learning rate = 0.1.

The following four measures were used to compare the classifier performances:

- *Sensitivity* (*True Positive Rate*)—the probability of a positive test result, conditioned on the individual sample being a real positive in histological analysis;

(1) $$Sn=\frac{TP}{TP+FN}$$

- *Accuracy* (*ACC*)—the percentage of correct predictions (both true positives and true negatives according to histological analysis) of the total number of samples;

(2) $$ACC=\frac{TP+TN}{TP+TN+FP+FN}$$

- *Positive Predictive Value* (*PPV* or *Precision*)—is the ratio between the true positives (*TP*) according to histological analysis and all positive instances (sum of true positives and false positives).

(3) $$PPV=\frac{TP}{TP+FP}$$

- *Negative Predictive Value* (*NPV*)—is the ratio between the true negatives (*TN*), using histology as reference, and all negative instances (sum of true negatives and false negatives).

(4) $$NPV=\frac{TN}{TN+FN}$$

Simulations were performed on a graphic workstation with an 18-core CPU running at 4.20 GHz capable of running a maximum of 36 threads in parallel (Intel^®^ Core™ i9-7980XE), a dedicated graphic card with 11 GB of DDR5X memory (GeForce GTX 1080 Ti Waterforce), and a volatile memory of 64 GB DDR4 quad channel running at 2133 MHz. The software application was designed using the MATLAB^®^ R2023a integrated development environment and the *Digital Image Processing* and *Deep Learning toolboxes*. The Digital Image Processing Toolbox was used for image manipulation and image processing, while the Deep Learning Toolbox was used for CNN-based classification. As CNNs are trained on a large image database, the computer GPU was used to increase the processing speed.

Several patches were extracted from endoscopic images of both CD and controls, sized 100 × 100 pixels. Among them, 70% were used for the training dataset, while 30% were used for testing. As the dataset was not large enough, a simpler train–validation split was used. Therefore, no cross-validation techniques were performed [27]. ML and DL algorithms were then consecutively applied to image samples and their diagnostic performance was compared to reference histology.

**Figure 5:**
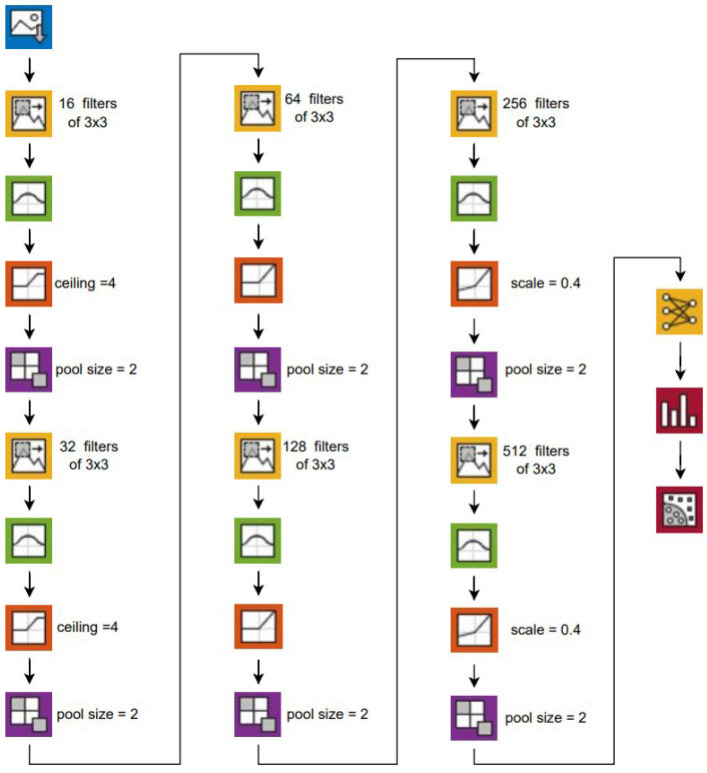
Layers of the DL algorithm.

Best DL classification results were obtained with convolutional neural network (CNN) layers as described in Figure 5. A CNN consists of three types of layers: an input layer, several hidden convolutional layers, and an output layer. Similar to visual cortex neurons, each convolutional layer processes the input data for its particular receptive field and then passes the result to the next layer. Convolutional layers also include pooling layers that reduce data size by combining the outputs of neuron clusters into a single neuron. The CNN uses the Adam (Adaptive Moment Estimation) optimizer with an initial learning rate of 10^−5^ to improve the accuracy and speed of the deep learning model by adjusting its parameters. It is an enhanced version of the more common stochastic gradient descent (SGD) algorithm used to update the neural weights of the CNN. Unlike traditional neural networks, CNNs share their weights and biases between neurons and layers, meaning that all neurons in hidden layers detect the same feature in various regions of an image.

## 3. Results

A total of 34 patients were included in the final analysis, with a median age of 43 years, 64.7% female, 18 confirmed with CD, and 16 controls. All CD patients had atrophic mucosa on duodenal biopsy samples and positive tissue transglutaminase antibodies, while non-CD controls had normal villous architecture and negative CD serology.

A number of 66 white light endoscopy (WLE) images were captured from the 18 CD patients and 16 control endoscopic images, 1 image from each non-CD control. After the automatic patch extraction process, we obtained 90 control patches and 477 CD patches.

Table 2 presents performances obtained for each patient group, using ML and DL algorithms. Sub-images (patches) were divided into two databases: training (397 patches) and testing (170 patches). CNN provided the best TP ratio of 142 correctly identified CD patches out of 143, as well as the best TN ratio of 25 control patches out of 27.

Using histology as the reference standard, the following diagnostic performances were obtained using the ML algorithm: WKNN—97.20% sensitivity, boosted trees—94.41% sensitivity, and bagged trees—98.60% sensitivity. A 99.30% sensitivity was obtained using the CNN. The global comparative results are shown in Table 3.

## 4. Discussion

CD diagnosis has evolved over the years, from the initial Interlaken criteria—consisting of mucosal lesion on the jejunal biopsy, which recovers on a gluten-free diet and relapses on re-challenge to gluten [28], to atrophic mucosal injury in the proximal small bowel as detected by upper gastrointestinal endoscopy. Fueled by the already validated experience from pediatrics with the ESPHGAN guidelines [4], and because of the several pitfalls associated with duodenal histology, interest towards a non-bioptic diagnosis of adult CD has grown considerably in the recent literature and has been approached in the most recent guideline update [29]. In this setting, we proposed a novel computer-based algorithm, which accurately detected VA and can aid in CD diagnosis.

We evaluated the diagnostic accuracy of our algorithm in atrophic (Marsh 3) versus non-atrophic (Marsh 0-1) mucosa from endoscopy-captured images, keeping in mind that VA markers are seen with all stages of atrophy (Marsh 3a–c) and that the relevance of the subdivision of Marsh 3 stages is being questioned by some authors [30]. On the other hand, different VA markers might be more evident or subtle with different sub-stages of Marsh 3, from a to c; thus, future studies should consider using AI algorithms to differentiate between different stages of CD disease. Due to the fact that the transition between different stages of the CD is continuous, there is no hard visual discrimination between those classes. Therefore, some classification errors might appear when detecting adjacent classes (e.g., Marsh 3a and 3b or Marsh 3b and 3c).

In the current research, we assessed the diagnostic accuracy of computer-based algorithms in detecting VA on images captured during endoscopy. While the CNN algorithm had a very small miss rate, if backed up by high titer serology, it might be sufficient to justify a lifelong diagnosis and subsequently restrictive diet. Among the counter-arguments of using a non-bioptic strategy, some have debated that adherence to a diet might be influenced if not for a strong argument such as histologic evidence of diseased bowel mucosa [31].

On the other hand, there is a good premise for a computer-aided diagnosis of VA during the endoscopic examination, considering the impact of already validated techniques in improving the detection of mucosal changes, such as increasing adenoma detection rate in AI-assisted colonoscopy.

The duodenal examination is frequently overlooked and mostly focused on the pathology of the papilla and peptic changes in the duodenal bulb. However, the duodenum is one of the landmarks set for photo documentation during endoscopy; moreover, included as a quality indicator in guidelines [32], so each endoscopy could be an opportunity to screen for abnormalities of the duodenal mucosal architecture. There is a need for increasing awareness among endoscopists to carefully inspect the duodenum during an upper gastrointestinal examination, in order to maximize visual or computer-aided detection of VA markers. Also, adhering to guidelines [33] with respect to examination time would make the endoscopist more thorough in visualizing and recognizing duodenal mucosal changes, which may be the sole manifestation of CD.

While there is an increasing debate in the current literature with regard to a biopsy-avoiding diagnosis for adult CD, we should differentiate between a non-biopsy and a non-endoscopy diagnosis. While computer-based algorithms could provide a non-biopsy diagnosis, patients would still have to undergo the endoscopic examination to capture images and biopsy avoidance would be beneficial for patients exposed to bleeding risks or for patients unwilling to consent for biopsy sampling. On the other hand, a non-endoscopy diagnosis, based on serology only (as the current criteria used in pediatric guidelines) would also account for adults unwilling or unable to undergo endoscopy. Moreover, as we already have validated algorithms for the detection of VA on capsule endoscopy and upper gastrointestinal endoscopy images, as on quantitative histology on biopsy slides [13,18], there is potential in using AI for real-time histological diagnosis of CD when using confocal laser endomicroscopy probes during the examination, which would obviate the need to take biopsies in patients not willing to undergo biopsy sampling or at hemorrhagic risk.

Another point to consider about AI algorithms is the computing speed. While some studies have proposed complex algorithms on capsule endoscopy images [13], more simple ones would provide high processing speed, much needed in order to promptly detect mucosal changes in the duodenum, which is subject to peristalsis, respiratory movements, and circulatory pulsations.

In addition to the detection of VA during an endoscopic examination carried out for non-CD indication, which would drive the endoscopist to perform a biopsy and diagnose a clinically silent or inapparent CD, using real-time AI algorithms to detect duodenal mucosa abnormalities would also aid in targeting biopsies in the diseased areas, taking into account that VA can be patchy and random sampling might miss the diagnosis [34].

### Limits and Strengths

In this study, we assessed for VA in CD patients versus controls in endoscopy images captured from the duodenum. However, it is well known that VA can be patchy and sometimes confined only to the duodenal bulb (the so-called “ultra-short CD”) [35,36,37]. The selection of images could represent a bias in the current study, but this was meant to be a proof-of-concept study and our results should be validated in larger cohorts, also on duodenal bulb images. Although we used images captured during a certain time point during the endoscopic examination, aiming at visualizing the duodenum in the same instance, there was no standardization with regard to angle or proximity to the mucosa in the captions selected for analysis. This selection bias, which can also mean using unrepresentative images because of the patchiness of the disease, would be eliminated if AI algorithms were incorporated into endoscopy software and used in real-time during the examination, as the currently validated applications for adenoma detection during colonoscopy [38]. Also, considering that endoscopy could represent a case-finding tool to detect CD, not analyzing images from the duodenal bulb is also a limit, which would miss about one in ten CD patients [39,40]. However, caution is recommended when detecting VA in the duodenal bulb, as morphological injury is common in the bulb in the absence of CD [41]; although this has been reported for histology-detected VA, this may be extrapolated to endoscopically detected VA.

Also, there is a proportion of CD patients with normally appearing duodenum that might be missed by a computer-processing technique of images, if they would not undergo biopsies and histological assessment of mucosa. In some cases, the use of advanced endoscopic techniques such as chromoendoscopy or water immersion might better delineate subtle mucosal changes [42,43], which would otherwise be missed on white light endoscopy, and computer-aided algorithms should also be tested on enhanced images captured during chromoendoscopy. Considering the high diagnostic performance of AI, further research should specifically look at patients with misinterpreted normal endoscopic examinations and missed CD, in fact with slightly diseased mucosa, and the impact of accurately detecting VA using quantitative computer-aided image processing.

On the other hand, VA can occur in other enteropathies besides CD [44,45]—Table 4—so the detection of VA using AI is not equivalent to CD diagnosis. CD-specific serology and particular histological criteria are used for the wide differential of VA.

Another potential drawback of using image processing techniques in recognition of pathological mucosal patterns is represented by artifacts—while in colonoscopy the endoscopists are struggling with fecal residues, which can be diminished with optimized bowel preparation, in the duodenum the visibility can be impaired by foaming and bubbles, but this can also be improved with premedication such as pronase, N-acetylcysteine, or simethicone [46,47]. To counteract the situations where pre-endoscopy preparation with such antifoaming and mucolytic agents is not used, the algorithm we proposed eliminates artifacts and extracts clean image frames for analysis of VA markers.

The biggest limitation of deep learning models comes from the fact that they learn through observations, which means that they perform only with the types of data which they were trained with. If a user has a small amount of data or data comes from one specific source that is not necessarily representative of the broader functional area, the models will not learn in a way that is generalizable [48]. In this respect, there is a need to validate our algorithm on large-scale dataset images, captured from CD patients, and non-CD controls but also on gluten-free diet-treated CD patients with mucosal healing.

To our knowledge, this is the first study to report a quantitative analysis of endoscopy images in diagnosing VA. Further validation of our algorithm is required in future studies with large cohorts of patients, and large datasets of images, maybe using captures from different endoscopy providers and also testing it on chromoendoscopy images. Also, future work should focus on the impact of using AI techniques for CD diagnosis against clinically relevant endpoints such as improvements in the CD diagnostic rate at the populational level or among certain high-risk groups and detection of clinically inapparent or previously missed CD.

## 5. Conclusions

Computer-aided detection of VA by the processing of images captured during upper gastrointestinal endoscopy is feasible and could be implemented as a case-finding strategy for CD. A layered CNN algorithm analyzing image patches from the duodenum of newly diagnosed CD patients compared to non-CD controls detected VA with a 99.30% sensitivity and a 98.61% positive predictive value. ML techniques tested also showed good diagnostic performance, but slightly lower than the DL technique. The incorporation of such AI techniques into endoscopy equipment and applying it in real-time during duodenal inspection can improve the detection of unsuspected CD.

Using the algorithm described in our research, further studies should test its diagnostic accuracy in Marsh 3 subtypes and also in gluten-free diet-treated patients. In order to enhance classification results by extracting the best capabilities from various individual classifiers, the use of decisional fusion can be envisaged.

## Figures and Tables

**Table 1:** Endoscopic markers of VA.

Atrophy of mucosa with prominent submucosal vascular pattern; Mucosal fissures or grooves, with mosaic or “cracked-mud” appearance; Nodularity of the mucosa; Scalloping of Kerckring folds; Reduction or loss of folds.

**Table 2:** Performances of ML and DL algorithms for each group (CD and control).

Class	Samples	Images	Patches	WKNN	Boosted Trees	Bagged Trees	CNN
CD	18	66	477	139/143	135/143	141/143	142/143
Control	16	16	90	14/27	13/27	13/27	25/27

**Table 3:** Global performances of ML and DL algorithms.

Algorithm	Technique	Sensitivity	Accuracy	PPV	NPV
ML	WKNN	97.20%	90.00%	91.45%	77.78%
	Boosted trees	94.41%	87.06%	90.60%	61.90%
	Bagged trees	98.60%	90.59%	90.97%	86.67%
DL	CNN	99.30%	98.24%	98.61%	96.15%

**Table 4:** Non-celiac villous atrophy causes.

Infectious (Giardia, Helicobacter pylori, Whipple’s disease, viruses); Common variable immune deficiency; Autoimmune enteropathy; Inflammatory bowel disease; Eosinophilic gastroenteritis; Peptic duodenitis; Small intestinal bacterial overgrowth; Drug-induced enteropathy (olmesartan, non-steroidal anti-inflammatory drugs).

## Data Availability

The dataset is available from the corresponding author.

## Abbreviations

The following abbreviations are used in this manuscript:

ACC	Accuracy
Adam	Adaptive Moment Estimation
AI	Artificial Intelligence
CD	Celiac Disease
CNN	Convolutional Neural Network
DL	Deep Learning
FN	False Negative
FP	False Positive
ML	Machine Learning
NPV	Negative Predictive Value
PPV	Positive Predictive Value
SGD	Stochastic Gradient Descent
TN	True Negative
TP	True Positive
VA	Villous Atrophy
WKNN	Weighted K-Nearest Neighbors
WLE	White Light Endoscopy
